# Hybrid feature engineering of medical data via variational autoencoders with triplet loss: a COVID-19 prognosis study

**DOI:** 10.1038/s41598-023-29334-0

**Published:** 2023-02-17

**Authors:** Mahdi Mahdavi, Hadi Choubdar, Zahra Rostami, Behnaz Niroomand, Alexandra T. Levine, Alireza Fatemi, Ehsan Bolhasani, Abdol-Hossein Vahabie, Stephen G. Lomber, Yaser Merrikhi

**Affiliations:** 1 Department of Medicine, Shahid Beheshti University of Medical Sciences, Tehran, Iran; 2 Department of Physiology, McGill University, 3655 Promenade Sir William Osler, Montreal, QC H3G1Y6 Canada; 3 Department of Psychology, University of Western Ontario, London, Ontario N6A 3K7 Canada; 4 Department of Internal Medicine, Shohadaye Tajrish Hospital, Shahid Beheshti University of Medical Sciences, Tehran, Iran; 5 Department of Physics, University of Isfahan, Isfahan, 81746-73441 Iran; 6 Cognitive Systems Laboratory, Control and Intelligent Processing Center of Excellence (CIPCE), School of Electrical and Computer Engineering, College of Engineering, University of Tehran, Tehran, Iran; 7 Department of Psychology, Faculty of Psychology and Education, University of Tehran, Tehran, Iran; 8 Pasargad Institute for Advanced Innovative Solutions (PIAIS), Tehran, Iran

**Keywords:** Respiratory tract diseases, Medical research

## Abstract

Medical machine learning frameworks have received much attention in recent years. The recent COVID-19 pandemic was also accompanied by a surge in proposed machine learning algorithms for tasks such as diagnosis and mortality prognosis. Machine learning frameworks can be helpful medical assistants by extracting data patterns that are otherwise hard to detect by humans. Efficient feature engineering and dimensionality reduction are major challenges in most medical machine learning frameworks. Autoencoders are novel unsupervised tools that can perform data-driven dimensionality reduction with minimum prior assumptions. This study, in a novel approach, investigated the predictive power of latent representations obtained from a hybrid autoencoder (HAE) framework combining variational autoencoder (VAE) characteristics with mean squared error (MSE) and triplet loss for forecasting COVID-19 patients with high mortality risk in a retrospective framework. Electronic laboratory and clinical data of 1474 patients were used in the study. Logistic regression with elastic net regularization (EN) and random forest (RF) models were used as final classifiers. Moreover, we also investigated the contribution of utilized features towards latent representations via mutual information analysis. HAE Latent representations model achieved decent performance with an area under ROC curve of 0.921 (±0.027) and 0.910 (±0.036) with EN and RF predictors, respectively, over the hold-out data in comparison with the raw (AUC EN: 0.913 (±0.022); RF: 0.903 (±0.020)) models. The study aims to provide an interpretable feature engineering framework for the medical environment with the potential to integrate imaging data for efficient feature engineering in rapid triage and other clinical predictive models.

## Introduction

The COVID-19 outbreak has become a major global health concern, causing significant mortality and morbidity^1^. Vaccination and prevention policies have played a significant role in containing the pandemic, however without fully approved treatment options, the mortality and morbidity rate of the disease in admitted patients, especially in patients with comorbidities and severe conditions, shows little decline^2,3^.

A COVID-19 diagnosis is mainly based on the combination of clinical symptoms, CT scan findings, RT-PCR, and immunoassay techniques^4^. However, predicting the prognosis trajectory of the disease by relying on clinical judgments is not easy for physicians to do. In other words, physicians are often unable to predict the disease prognosis trajectory from patients' clinical data until more severe stages, which often leads to ICU admission and the need for mechanical ventilation. Prediction of the mortality prognosis is paramount in preventing disease worsening and leading to mortality by providing necessary interventions and efficient resource allocation. The data based on the underlying pathophysiology of the disease, such as laboratory markers and clinical features, can be helpful for prognosis prediction^5^. Furthermore, the prognosis of COVID-19 is critical for local and national health administration centers in order to inform and provide essential policies for the management and control of the ongoing pandemic^6^. The possible underlying mechanism of the disease is mainly based on inflammation and the immune response of patients; however, the pathophysiology of SARS-CoV-2 pathogenicity has not been thoroughly explored^6^. Therefore, clinical manifestations and laboratory data, which reflect the underlying disease progression, could be applied to detect disease prognosis^7^.

Artificial Intelligence (AI) models are valuable tools for physicians and healthcare managers, able to detect complex patterns in large datasets and achieve high accurate prognosis accuracy^2^. Several machine learning models have been established to predict disease outcomes since the onset of the pandemic. These models have used various feature types, including demographic data, comorbidities, laboratory features, and imaging data, to enhance their models' accuracy in prediction. However, a validated model or scoring system applicable for measuring the patients' prognosis in the early stages of the disease is controversial^8^. A prominent issue in almost all medical machine learning scenarios, including COVID-19 prognosis prediction, is feature engineering and dimensionality reduction of the large available data. Autoencoders (AE) and Variational Autoencoders (VAE) have been widely used for dimensionality reduction in various fields^9,10^. The combination of Autoencoders with Triplet loss has allowed more efficient dimensionality reduction of image data^11^. However, few studies have investigated the potential of combined VAE and Triplet loss frameworks for dimension reduction of non-image medical data. The current study investigated the power of latent representations obtained from a hybrid autoencoder (HAE), which utilizes Mean Squared Error (MSE) and Triplet loss with a layer structure similar to VAE to forecast COVID-19 mortality using non-image medical data, including clinical and laboratory data.

## Materials and methods

### Study setting and population

The study protocol was approved by the Ethics Committee of Shahid Beheshti University of Medical Sciences (SBMU) and carried out following the Helsinki and SBMU guidelines and regulations. The retrospective data was retrieved from archived electronic medical records of 1625 patients with confirmed COVID-19 who were admitted to the Shohadaye Tajrish Hospital in Tehran, Iran, between March 15th, 2020, and June 15th, 2020.

The inclusion criteria were hospital admission due to confirmed SARS-Cov2 infection using a Real-Time PCR test according to the 5th Iranian COVID-19 guideline. Two physicians assessed each patient's medical information before approval by two infectious diseases and internal medicine experts. Furthermore, 151 patients were removed from the study: 19 patients with age under 18, 5 patients due to pregnancy or breastfeeding, 32 patients who received radically different treatment protocols (e.g., clinical trials), 14 patients who left the hospital against medical advice, four patients were referred to other treatment centers, and 77 patients were removed due to more than 20% missing data in their medical records. In the end, 1474 patients were included in the study. Informed consent was obtained from all patients and, if applicable, their legal guardians regarding patient data utilization for medical research upon admission. Patient data were anonymized before the data analysis phase.

For further assessment of pipeline generalizability, a separate public dataset from the Masih Daneshvari Hospital in Iran was also employed in the study^2^. In a similar prognosis prediction context, the dataset consisted of 492 patients and 37 features with a binarized outcome (expired vs. discharged).

### Variable definition

The initial data was categorized into three groups. Demographic and clinical data were extracted from patients' admission history, medical progress notes, and nursing notes. Comorbidities were extracted from the admission checklist and previous medical documents confirming the comorbidity. Cardiovascular diseases consisted of ischemic heart disease (IHD), arrhythmias, heart failure, and valvular dysfunctions. Airway diseases included obstructive and restrictive pulmonary diseases. Other comorbidities, including cancer, liver, and renal diseases, were confirmed by the hospital's physicians. Patients' vital sign data was extracted from admission history and nursing notes. During admission, blood pressure and pulse rate were measured using an electronic blood pressure patient monitoring device, the temperature was measured using a digital forehead thermometer, and the patients' oxygen saturation was measured in the room air without oxygen support using an electronic pulse oximeter. The laboratory data were extracted from the results of serial blood tests ordered by the physician during admission. All blood samples were obtained from venous blood and analyzed in the hospital's central laboratory. Referral results from other laboratories were excluded from the study. The collected features and their brief definitions are presented in supplementary table 1. The absolute neutrophil and lymphocyte counts were calculated by multiplying their percentage by the White Blood Cell (WBC) count. Qualitative troponin was obtained from quantitative troponin with a threshold of 0.02 for positive troponin. Partial Thromboplastin Time (PTT) results higher than 41 (displayed as ">41" in the lab data records) and PT higher than 6 (displayed as ">6" in the lab data records) were converted to 41 and 6, respectively. Features with more than 20% missing values were omitted from the analysis. Finally, 79 features, including 6 demographic and clinical features, 5 vital signs, 8 comorbidities, 2 habitual history features, and 58 laboratory features, were used as input features of the models (Supplementary Table 2). Indices of 0 and 1 in lab results (e.g., Red Blood Cell (RBC) 0 and RBC 1) denote the first and second laboratory tests that were obtained from patients. The first lab test battery was ordered upon patient's admission, and the second tests were often scheduled for 24 hours after admission, unless the physician deemed a test unnecessary, in which case, the test was not included in the second battery or was obtained at a different time.

### Data preprocessing and statistical analysis

The input features were imputed using the Multiple Imputation by Chained Equations (MICE) with lightgbm via the miceforest python library^12^. Briefly, MICE is a flexible method of dealing with missing data where the missing values are filled based on the observed values for a given data point and the observed relations in the data, creating multiple imputed datasets^13^. The current study used a k=5 for the number of created imputed datasets and used the mean of the resulting imputed datasets for further analysis steps. The isolation forest^14^ algorithm, a framework routinely used for anomaly and outlier detection, was utilized to remove prominent outliers from the imputed dataset with a contamination ratio of 0.01. The isolation forest algorithm identifies anomalous datapoints based on the distance from the node that isolates them and the starting node.

The normality of variable distributions was tested using the Kolmogorov–Smirnov test. Normal variables were displayed using mean ± standard deviation. The median with an interquartile range was used to display non-normal variables. T-test and Kruskal–Wallis test with a p-value threshold of 0.05 were utilized to detect significant differences between the two comparison groups in the normal and non-normal groups, respectively.

### Modelling

#### Autoencoders

Autoencoders (AEs) are state-of-the-art unsupervised deep learning frameworks with the main purpose of learning informative representations, often in lowered dimensions, from the input data to reconstruct them. These informative representations can then be used for various purposes, such as clustering and visualization^9^. Low-dimensional structures of real-world data are usually complex and cannot be completely decoded by routine dimensionality reduction techniques, such as PCA. In contrast, AEs have demonstrated an encouraging ability to learn meaningful representations from the data^15^. Towards this aim, encoder layers try to learn a function that maps the original input space into the latent space (R^n^ => R^k^), with $k < n$, and the decoder layers try to learn a function that reconstructs the original features from the latent representation (R^k^ => R^n^)^16^. An AE network aims to minimize a loss that is usually defined to be the L2 norm of the difference between the reconstructed and original values measured by the Mean Squared Error (MSE). Stemmed from the vanilla AEs, Variational Autoencoders (VAEs) are generative models that try to explain the data through the lens of probabilistic distributions. Briefly, given an observed dataset, **X**, a generative model, sometimes called a probabilistic decoder, is assumed for each datum **x**_**i**_. This distribution is conditioned upon the latent representation **z**_**i**_ and is governed by the parameter set **Θ**. On the other end of the network, given datum **x**_**i**_, an approximate encoder distribution is assumed over **z**_**i**_ that is directed by the parameter set **Φ**. The loss of a routine VAE is obtained by summing the MSE and Kullback–Leibler (KL) divergence losses, where the KL divergence tries to make the decoder and encoder distributions more similar. A common approach to approximating **Θ** and **Φ**, also adapted by the current paper, is assuming a multivariate Gaussian distribution for both, where the distribution of **Θ** is assumed to have a mean of 0 and an identity covariance matrix (*N*(**0**, **I**)) and the distribution of **Φ** is assumed to have a mean of **µ** and a covariance matrix of **Σ** (*N*(**µ**, **Σ**)). With this setting, the latent representation **z** is calculated by **z** = **Σ**ζ + **µ**; the ζ distribution is a source of noise that can enhance the model's generalizability and has a distribution of *N*(**0**, **I**). A more detailed explanation of AEs can be found in^9^. During initial exploratory steps, the vanilla VAE displayed poor performance and thus was excluded from further analysis steps. Instead, a modified VAE with MSE loss only was utilized for obtaining latent representation.

Triplet loss is a recent successful approach that enhances the performance of deep learning models by guiding the model toward learning useful latent representations using distance metrics. Mainly used for image and semantic tasks, the loss is defined using three sets of points; an anchor set, a positive set, and a negative set. For each anchor point in the learning batch, a corresponding positive point (same class) and a negative point (from a class other than anchor) are selected from the training set^11^. The loss with Euclidean distance is defined as:

$$L\left( {A,P,N} \right) = max\left(\| {f\left( A \right) - f\left( P \right)\|^{2} -\| f\left( A \right) - f\left( N \right)\|^{2} + \alpha ,0} \right)$$

where f is the latent embedding. A, P, and N are anchor, positive, and negative points, respectively. The α parameter is a margin between positive and negative pairs. The cost function sums all of the individual losses:

$$J = \mathop \sum \limits_{i = 1}^{M} L\left( {A^{i} ,P^{i} ,N^{i} } \right)$$

To investigate the predictive powers of latent representations obtained using Triplet (TL), a hybrid autoencoder (HAE) with representation layers similar to those of a VAE but with MSE and TL loss was utilized. The same layer characteristics (Fig. 2A) were used for HAE and modified VAE to obtain latent representations from raw inputs over 700 Epochs with a batch size of 128 and SELU as the activation function. To avoid overfitting, validation sets were utilized to assess the performance, and only save model parameters if the model passed an improvement tolerance threshold of 0.01. Tuning of the autoencoder hyperparameters was conducted using the Ray Tune library^17^. Ray Tune is a widely used hyperparameter tuning tool that utilizes updated hyperparameter tuning algorithms and can be integrated with various analytic libraries. The search dictionary consisted of the unit numbers in each layer, learning rate, and batch size. The SELU function was chosen in light of its beneficial characteristics, such as self-normalization and superior performance^18^. All AE models were implemented using PyTorch v1.10 and Cuda v11.3 on a GeForce GTX 1050 graphic card.

#### Classifier models

Raw features along with latent representations from the modified VAE and HAE models were used as input for classifier models**.** The input data were randomly split into stratified training and test sets. To alleviate the unbalanced data issue, upsampling and balanced class weights were utilized for training. Initially, the inputs were divided into training and test sets. The upsampling was performed after splitting the training and test sets to ensure the presence of a true test set, which was solely used for the final performance assessment. To perform classifications on representations and raw data, Logistic regression with Elastic Net regularization (EN) and random forest (RF) classifiers were utilized. Logistic regression and RF algorithms have been extensively used to analyze medical data, making them appropriate candidates for benchmarking purposes^19–22^. Furthermore, a beneficial characteristic of the Logistic regression with Elastic Net and random forest model is the presence of characteristics that act as inherent feature selections and modestly decrease overfitting. These characteristics can alleviate the impacts of features with low predictive information in high-dimensional feature spaces^23,24^.

To optimize classifier hyperparameters, Bayesian optimization with cross-validation using 5 stratified folds over the training set in a defined search space was utilized over 40 (EN) and 30 (RF) iterations. Weighted F1 (weighted based on the number of instances of each class) was used as the optimization metric. Classifier models were implemented using the scikit-learn library v1.0.2. Optimization was conducted via the scikit-optimize library v0.9. To obtain box plots of model metrics, all pipelines were executed for 50 iterations, and during each iteration, a fresh random train-test split was obtained. All models in an iteration were trained and tested using instances separated with similar random states to avoid bias.

#### Feature importance

Feature coefficients from the logistic regression model with L1 regularization were obtained as representations of the predictive power of features. Moreover, to untangle the latent representations from HAE models, the normalized mutual information (MI) between the representations and raw input features was calculated^25^. This approach allowed us to peek inside the model's black box to investigate the contribution of features to the formation of latent representations.

#### Metrics

Metrics used to evaluate the models' performances on the test set were as follows; 1) Area under the ROC curve (AUC), 2) Precision 3) Recall 4) F1-score 5) Bayesian optimization time, 6) Balanced accuracy, and 7) Average precision. Due to the significance of detecting high-risk patients and avoiding interpretation biases, reported precision, recall, and F1 scores are from the expired class, as the models performed quite well on the discharged class (high precision, recall, and F1 scores). Average precision summarizes the precision-recall curve by averaging the weighted precisions achieved at each threshold, and balanced accuracy tries to deal with unbalanced data by reporting the average of each class's recall^26^. The Bayesian optimization time was recorded from the start of the optimization's iterations (40 for EN and 30 for RF) until the end of optimization. All modeling frameworks were implemented in the Python v3.8 environment. The plots were created via MATLAB R2019b using the exported results from the modeling steps.

### Ethics

The study protocol was approved by the Ethics Committee of Shahid Beheshti University of Medical Sciences (SBMU) and carried out following the Helsinki and SBMU guidelines and regulations.

## Results

Data from 1474 individuals were used for model training. The median age for the study population was 58 (30). Among the study population, 782 (53%) were male, and 692 (47%) were female. Regarding prognosis outcome, 1214 (82.4%) patients were discharged after completion of the treatment course, and 260 (17.6%) patients expired during their treatment course. Cough (50.8%), shortness of breath (49.6%), and fever (38.2%) were the three most frequent symptoms among patients. Furthermore, hypertension (33.0%), diabetes mellitus (23.1%), and cardiovascular diseases (19.7%) were the most frequent comorbidities (Table 1). The detailed descriptive characteristics of laboratory data in both discharged and expired patients are displayed in Table 2.

**Table 1:** Demographic and clinical features of the expired and discharged patients.

Characteristics	Discharged	Expired	Total	*P *value	AUC
Age, median (Q_3_-Q_1_)	55.00 (28.00)	72.00 (21.00)	58.00 (30.00)	<0.001	0.733
Sex				0.599	0.491
Male (%)	53.33	51.54	53.02		
Female (%)	46.67	48.46	46.98		
Clinical symptoms on admission					
Shortness of breath (%)	48.48	55.00	49.63	0.056	0.533
Sputum (%)	23.13	13.85	21.49	0.001	0.454
Hemoptysis (%)	5.51	1.92	4.88	0.015	0.482
Chills (%)	36.05	20.77	33.36	<0.001	0.424
Cough (%)	52.84	41.15	50.78	0.001	0.442
Fever (%)	39.09	33.85	38.17	0.114	0.474
Headache (%)	15.80	7.69	14.37	0.001	0.459
Sore throat (%)	6.83	1.92	5.97	0.002	0.475
Dizziness (%)	12.43	2.31	10.64	<0.001	0.449
Stomachache (%)	8.15	5.38	7.66	0.128	0.486
Nausea (%)	23.62	11.92	21.56	<0.001	0.442
Vomit (%)	17.78	14.23	17.15	0.169	0.482
Diarrhea (%)	13.91	11.54	13.49	0.310	0.488
Body Pain (%)	34.90	22.31	32.68	<0.001	0.437
Comorbidities					
Diabetes mellitus (%)	20.74	34.23	23.12	<0.001	0.567
Hypertension (%)	30.62	44.23	33.02	<0.001	0.568
Cardiovascular disease (%)	18.35	25.77	19.66	0.006	0.537
Airway disease (%)	6.67	7.69	6.85	0.552	0.505
CVA (%)	3.29	13.85	5.15	<0.001	0.553
Cancer (%)	4.53	8.46	5.22	0.010	0.520
Renal (%)	5.68	11.54	6.71	0.001	0.529
Liver (%)	0.99	1.15	1.02	0.808	0.501
Vital signs					
Blood pressure max, median (Q_3_-Q_1_)	120.00 (20.00)	113.40 (28.00)	120.00 (20.00)	0.050	0.462
Blood pressure min, median (Q_3_-Q_1_)	75.00 (10.00)	70.00 (20.00)	75.00 (10.00)	0.003	0.443
Pulse rate, median (Q_3_-Q_1_)	75.00 (12.00)	70.00 (20.00)	84.20 (13.00)	0.001	0.567
Respiratory rate, median (Q_3_-Q_1_)	18.00 (3.00)	19.60 (5.60)	18.00 (3.00)	<0.001	0.588
SpO_2,_ median (Q_3_-Q_1_)	91.00 (8.00)	84.00 (15.00)	90.00 (9.00)	<0.001	0.270
Drug history					
Corticosteroid (%)	2.80	4.23	3.05	0.223	0.507
Chemotherapy drugs (%)	0.91	3.08	1.29	0.005	0.511
Immunosuppressant drugs (%)	2.63	1.15	2.37	0.155	0.493
Habitual history					
Smoker (%)	5.35	6.15	5.49	0.605	0.504
Addiction (%)	7.41	6.15	7.19	0.477	0.494
Alcohol (%)	1.56	2.69	1.76	0.209	0.506

Continuous variables are described by median and inter quartile range. Categorical variables are described by percent. The AUC of individual variables was calculated using simple Logistic Regression. CVA: Cerebrovascular Accident. SpO_2_: Blood oxygen saturation. AUC: Area Under ROC Curve.

**Table 2:** Laboratory characteristics of expired and discharged patients.

Characteristics	Discharged	Expired	Total	P-value	AUC
Complete blood count (CBC)					
* WBC_0, median (Q*_*3*_*-Q*_*1*_*)*	8.10 (5.80)	10.80 (2.60)	10.00 (5.80)	<0.001	0.713
* WBC_1, median (Q*_*3*_*-Q*_*1*_*)*	7.26 (5.82)	11.60 (3.88)	7.96 (6.26)	<0.001	0.734
* Neut_0, median (Q*_*3*_*-Q*_*1*_*)*	70.00 (19.00)	80.00 (15.50)	70.40 (17.00)	<0.001	0.722
* Neut_1, median (Q3-Q1)*	75.00 (14.00)	85.00 (11.60)	77.00 (15.60)	<0.001	0.737
* Lymph_0, median (Q*_*3*_*-Q*_*1*_*)*	12.00 (10.00)	10.00 (0.00)	10.00 (8.00)	<0.001	0.295
* Lymph_1, median (Q*_*3*_*-Q*_*1*_*)*	16.00 (14.40)	10.00 (2.00)	15.00 (12.00)	<0.001	0.260
* Mono_0, median (Q*_*3*_*-Q*_*1*_*)*	2.00 (0.60)	2.00 (1.00)	2.00 (0.80)	<0.001	0.383
* R_B_C_0, median (Q*_*3*_*-Q*_*1*_*)*	4.02 (0.87)	3.61 (1.23)	3.96 (0.97)	<0.001	0.363
* R_B_C_1, median (Q*_*3*_*-Q*_*1*_*)*	4.19 (0.94)	3.80 (1.25)	4.12 (0.97)	<0.001	0.375
* HGB_0, median (Q*_*3*_*-Q*_*1*_*)*	11.50 (2.20)	10.50 (1.90)	11.30 (2.20)	<0.001	0.376
* HGB_1, median (Q3-Q1)*	11.90 (2.40)	11.00 (2.30)	11.80 (2.50)	<0.001	0.382
* HCT_0, median (Q*_*3*_*-Q*_*1*_*)*	33.20 (6.90)	30.05 (11.03)	32.80 (7.58)	<0.001	0.387
* HCT_1, median (Q*_*3*_*-Q*_*1*_*)*	34.40 (7.10)	31.44 (10.80)	34.16 (7.80)	<0.001	0.395
* PLT_0, median (Q*_*3*_*-Q*_*1*_*)*	173.00 (72.20)	140.10 (83.50)	169.00 (78.00)	<0.001	0.359
* PLT_1, median (Q*_*3*_*-Q*_*1*_*)*	193.00 (83.00)	154.50 (92.75)	188.40 (86.60)	<0.001	0.337
* MCV_0, median (Q*_*3*_*-Q*_*1*_*)*	79.63 (7.32)	80.76 (9.16)	79.77 (7.58)	0.047	0.539
* MCV_1, median (Q3-Q1)*	81.70 ((6.72)	81.95 (9.12)	81.70 (7.20)	0.468	0.514
* MCH_0, median (Q*_*3*_*-Q*_*1*_*)*	27.10 (2.41)	26.85 (2.79)	27.05 (2.52)	0.021	0.455
* MCH_1, median (Q3-Q1)*	27.83 (2.46)	27.29 (3.02)	27.73 (2.60)	<0.001	0.432
* MCHC_0, median (Q*_*3*_*-Q*_*1*_*)*	32.79 (2.20)	31.68 (2.50)	32.53 (2.30)	<0.001	0.328
* MCHC_1, median (Q3-Q1)*	33.50 (2.29)	32.38 (2.54)	33.28 (2.35)	<0.001	0.321
* ESR_0, median (Q*_*3*_*-Q*_*1*_*)*	18.00 (15.80)	23.00 (23.00)	18.60 (17.00)	<0.001	0.597
* CRP quantitative_0*	12.20 (14.50)	17.39 (19.85)	12.90 (16.04)	<0.001	0.620
Coagulation					
* PT_0, median (Q*_*3*_*-Q*_*1*_*)*	13.00 (0.68)	13.00 (1.59)	13.00 (0.70)	<0.001	0.585
* PTT_0, median (Q*_*3*_*-Q*_*1*_*)*	34.40 (6.00)	36.00 (18.75)	35.00 (7.00)	<0.001	0.593
* INR_0, median (Q*_*3*_*-Q*_*1*_*)*	1.00 (0.08)	1.00 (0.20)	1.00 (0.10)	<0.001	0.584
Biochemistry					
* BUN_0, median (Q*_*3*_*-Q*_*1*_*)*	13.00 (7.00)	23.00 (31.75)	14.00 (10.00)	<0.001	0.723
* BUN_1, median (Q*_*3*_*-Q*_*1*_*)*	16.00 (8.20)	27.00 (29.00)	17.00 (10.80)	<0.001	0.739
* Cr_0, median (Q*_*3*_*-Q *_*1*_*)*	0.90 (0.32)	1.06 (0.72)	0.91 (0.36)	<0.001	0.594
* Cr_1, median (Q*_*3*_*-Q*_*1*_*)*	1.00 (0.32)	1.17 (1.03)	1.01 (0.38)	<0.001	0.608
* AST_0, median (Q*_*3*_*-Q*_*1*_*)*	32.38 (21.52)	41.10 (60.81)	33.20 (24.72)	<0.001	0.603
* ALT_0, median (Q*_*3*_*-Q*_*1*_*)*	25.80 (22.60)	25.90 (34.36)	25.80 (24.10)	0.836	0.496
* ALkP_0, median (Q*_*3*_*-Q*_*1*_*)*	197.40 (88.00)	210.00 (145.25)	199.60 (95.00)	0.010	0.551
* LDH_0, median (Q*_*3*_*-Q*_*1*_*)*	471.00 (212.00)	774.50 (598.50)	496.00 (282.60)	<0.001	0.753
* CPK_0, median (Q*_*3*_*-Q*_*1*_*)*	133.00 (154.00)	135.50 (356.00)	133.40 (170.00)	0.008	0.552
* BS_0, median (Q*_*3*_*-Q*_*1*_*)*	122.00 (39.80)	130.00 (49.45)	123.00 (42.00)	0.051	0.539
* CKMB_0, median (Q*_*3*_*-Q*_*1*_*)*	21.80 (17.00)	25.00 (36.15)	22.00 (20.00)	<0.001	0.576
Electrolyte					
* Na_0, median (Q*_*3*_*-Q*_*1*_*)*	135.60 (4.96)	135.00 (6.97)	135.18 (4.96)	0.001	0.439
* Na_1, median (Q*_*3*_*-Q*_*1*_*)*	138.00 (4.82)	136.00 (5.67)	137.72 (4.80)	<0.001	0.431
* K_0, median (Q*_*3*_*-Q*_*1*_*)*	3.70 (0.40)	3.60 (0.70)	3.7090.40)	<0.001	0.417
* K_1, median (Q*_*3*_*-Q*_*1*_*)*	3.95 (0.50)	3.80 (0.79)	3.92 (0.50)	<0.001	0.419
Blood gas test					
* PH_0*, median (Q_3_-Q_1_)	7.28 (0.08)	7.17 (0.17)	7.27 (0.10)	<0.001	0.217
* PH_1, median (Q3-Q1)*	7.32 (0.07)	7.24 (0.13)	7.31 (0.08)	<0.001	0.205
* PCO2_0*, median (Q_3_-Q_1_)	40.00 (10.50)	35.60 (13.90)	39.36 (11.00)	<0.001	0.390
* PCO2_1*, median (Q_3_-Q_1_)	44.60 (11.00)	38.65 (13.29)	43.80 (12.20)	<0.001	0.360
* PO2_0*, median (Q_3_-Q_1_)	36.50 (77.20)	100.15 (87.48)	38.24 (79.30)	<0.001	0.594
* PO2_1*, median (Q_3_-Q_1_)	34.00 (20.06)	36.00 (81.76)	34.30 (23.62)	0.021	0.546
* HCO3_0*, median (Q_3_-Q_1_)	21.60 (5.50)	17.25 (6.23)	21.10 (6.00)	<0.001	0.262
* HCO3_1*, median (Q_3_-Q_1_)	23.76 (5.14)	19.00 (6.10)	23.20 (5.80)	<0.001	0.250
* BE_0*, median (Q_3_-Q_1_)	0.00 (1.20)	− 0.30 (5.48)	0.00 (1.48)	<0.001	0.383
* BE_1, median (Q3-Q1)*	− 0.10 (2.60)	− 1.40 (8.01)	− 0.28 (2.76)	<0.001	0.336
* O2sat_0*, median (Q_3_-Q_1_)	42.60 (23.40)	39.80 (27.58)	42.22 (24.30)	0.032	0.458
* O2sat_1*, median (Q_3_-Q_1_)	58.00 (23.24)	51.89 (25.73)	57.20 (24.26)	<0.001	0.428

AUC: Area under ROC curve, WBC: white blood cell, Neut: neutrophils, Lymph: lymphocytes, Mono: monocytes, RBC: red blood cell, HGB: hemoglobin, HCT: hematocrit, PLT: platelet, MCV: mean corpuscular volume, MCH: mean corpuscular hemoglobin, MCHC: mean corpuscular hemoglobin concentration, ESR: erythrocyte sedimentation rate, CRP: C-reactive protein, PT: prothrombin time, PTT: partial thromboplastin time, INR: international normalized ratio, BUN: blood urea nitrogen, Cr: creatinine, AST: aspartate transaminase, ALT: alanine aminotransferase, ALKP: alkaline phosphatase, LDH: lactate dehydrogenase, CPK: creatine phosphokinase, BS: blood sugar, CKMB: creatine kinase MB, Na: sodium, K: potassium, BE: base excess.

### Individual raw features and principal components contain limited predictive information

**Figure 1:**
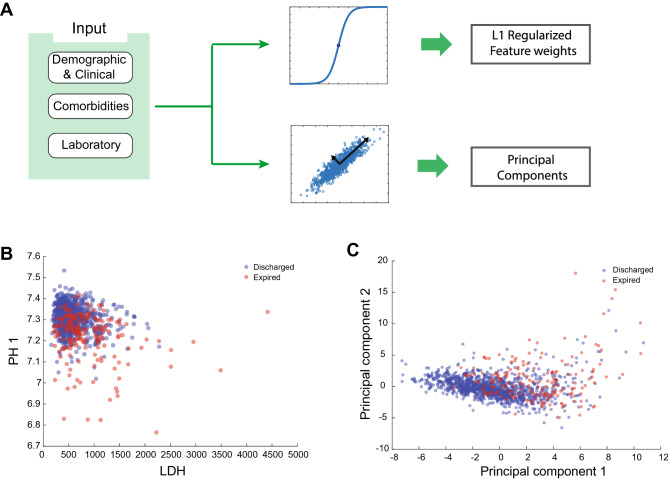
Predictive information content of principal components and informative features. (**A)** Input data were transformed into principal component space to obtain components with a high degree of variance explanations. To obtain informative features, logistic regression with L1 regularization was used to obtain sorted feature weights. (**B)** LDH and PH1 were selected based on their high absolute predictive weight value. Data points are entangled and hard to distinguish. **C)** Principal components 1 and 2 from the PCA analysis. In the PC space, the data points, similar to section B, are convoluted.

An initial feature exploration was conducted using logistic regression with L1 regularization to uncover features with high prognosis predictive powers. Furthermore, Principal Component Analysis (PCA) was also implemented to find principal components which explained significant variance in the data (Fig. 1A). As an unsupervised linear dimensionality reduction method, PCA is often used for data inspection to observe potential lower-dimensional structures in the data. Although the first two PCs explained about 20% of the data's variance, the variance explanation of PCs quickly dropped, and the rest of the variance was distributed between many features (See Supplementary Figure 1). When the data points were visualized using the first two principal components and two raw features with high predictive powers, the points were significantly intertwined, and no clear visual separation boundary was observable between expired and discharged patients (Fig. 1B, C). These results revealed that a few high-impact raw features and principal components were not, by themselves, informative enough to untangle the data points.

### HAE latent representations encompass significant predictive powers

**Figure 2:**
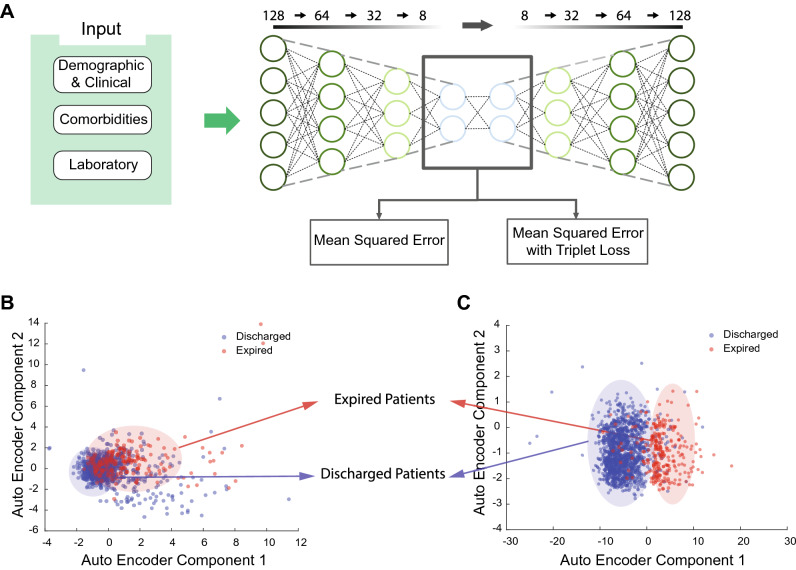
Autoencoder model overview and information content of principal components of AE representations. **(A)** The hybrid Autoencoder model (HAE) consisted of average and standard deviation representation layers along with Mean Squared Error (MSE) and Triplet (TL) loss. Two separate AE models were used to compare performance; a vanilla AE with one representation layer and a modified variation AE (VAE) with mean and standard deviation representation layers. Both models utilized MSE loss. **(B)** First and second principal components from the modified VAE model. The data points are convoluted and hard to distinguish. (**C)** First and second principal components from the HAE model. Expired and discharged patients have formed two distinct representation clusters in the PC subspace.

Next, we opted to evaluate the partitioning powers of the latent representations from the modified VAE and HAE models. The modified VAE model was optimized using MSE loss only, and the HAE model was optimized via MSE and TL losses. The representations were acquired from the middle mean and variance layers (Fig. 2A). PCA analysis of latent representations revealed an interesting result; no clear, distinct distribution of data points was observable in the PCs from the modified VAE model (Fig. 2B). In contrast, PCs from the HAE model's latent representations displayed two visually distinct clusters denoting the expired and discharged groups (Fig. 2C).

### HAE latent representations provide reasonable prognosis classification performance

**Figure 3:**
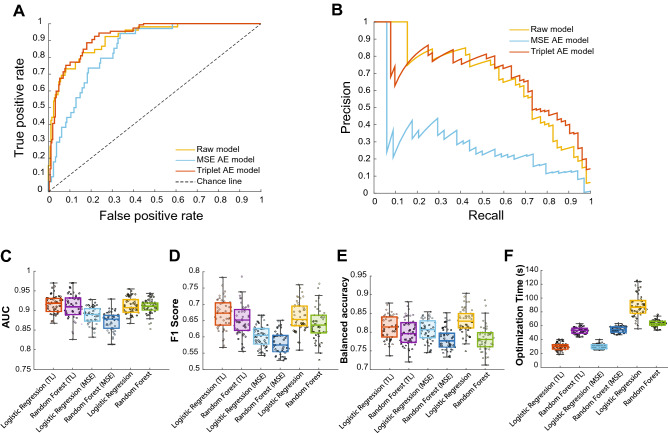
Classification performance of raw (full) features along with HAE and modified VAE representations. **(A)** ROC and (**B)** precision-recall curves of the classifier. The HAE model had a performance on par with the full model. The performance of the modified VAE model, however, was suboptimal compared to the other two models. (**C)** AUC box plots of the classification models. The AUC of the HAE model was close to the raw model and slightly better than the modified VAE model. (**D)** F1 score box plots of the classification models. The F1 score of the HAE model was close to that of the raw model and prominently better than the modified VAE model. (**E)** Balanced accuracy box plots of the classification models. The balanced accuracy of the HAE model was on-par with the raw model. **(F)** Bayesian optimization time of the classification models. Compared with the full model, the HAE and modified VAE models required significantly less time to be evaluated and optimized by the Bayesian optimizer. The optimization search spaces were similar for all three groups of input features. For panels C, D, E, and F, 50 iterations of training, optimization, and testing were utilized. TL: Models trained with representations from the HAE model; MSE: Models trained with representations from the modified VAE model.

After observing the results from the previous section, we aimed to see how the latent representations from AE models will perform for predicting patients' prognoses using common classification frameworks. Classifiers trained on raw features were employed for base comparison. The EN framework revealed that the models trained using the latent representations of the HAE models performed quite well (AUC: 0.921 (±0.027)), as seen in the ROC (Fig. 3A) and precision-recall (Fig. 3B) curves. The modified VAE, however, showed markedly lower performance compared with the models trained with raw and latent HAE features. Similar results were observed with the RF model (Supplementary Figure 2).

To further evaluate the performance of the models and avoid the potential bias effects of a single train-test split, we used several performance metrics in an iterative approach. To this aim, in each iteration, all pipelines were executed using a fresh train-test split. The results confirmed the previous findings; i.e., the classifiers using the HAE latent representations performed relatively well, with AUCs and F1 scores slightly better than those of the raw model (Fig. 3C–E; Table 3). Moreover, the HAE model also displayed good average precision (0.748 (±0.050)) (Supplementary Figure 3A) compared with the raw models. The EN model trained with HAE's representations appeared to be more balanced with respect to precision and recall (Supplementary Figure s3B,C) scores even though the f1 scores were close to those of the raw model (Table 3). An interesting finding was that although the modified VAE model displayed good AUC scores (Fig. 3C), it was biased, which was indicated by the reduced f1 score (Fig. 3D), poor precision (Supplementary Figure s3B), and increased recall scores (Supplementary Figure s3C). These results show that simply judging a model's performance based on one metric, such as AUC, only allows for limited deductions and can lead to fallacious deductions. Finally, although the performance of the HAE model was close to those of the raw model, the time required for Bayesian optimization of the classifier algorithm was significantly reduced (Fig. 3F). Similar to the results from the main dataset, results from the external validation revealed that classifiers using HAE latent representations provided decent classification performance compared with raw features and latent representations from the modified VAE model (Supplementary Figure 4, Supplementary Table 3). Combined with the ability of AEs to be implemented as online learning frameworks, this approach can markedly reduce the time required for retraining and optimizing a mounted classifier that uses the latent representations with respect to new data batches. In summary, latent representations from the HAE model provided better, balanced predictive performances while also reducing the Bayesian optimization time of classifiers.

**Table 3:** Performance metrics of classifier models.

Metric	EN + HAE	RF + HAE	EN + modified VAE	RF + modified VAE	Raw EN	Raw RF
AUC	0.921 (±0.027)	0.910 (±0.036)	0.880 (±0.022)	0.872 (±0.023)	0.913 (±0.022)	0.903 (±0.020)
Time(s)	29.49 (±5.08)	52.18 (±4.84)	29.67 (±3.36)	53.92 (±4.26)	88.65 (±16.38)	63.00 (±5.10)
F1	0.671 (±0.039)	0.653 (±0.042)	0.603 (±0.033)	0.581 (±0.034)	0.660 (±0.045)	0.628 (±0.046)
Precision	0.610 (±0.052)	0.633 (±0.061)	0.492 (±0.032)	0.483 (±0.034)	0.567 (±0.051)	0.624 (±0.0483)
Recall	0.764 (±0.064)	0.693 (±0.073)	0.783 (±0.063)	0.732 (±0.064)	0.784 (±0.060)	0.640 (±0.065)
Average precision	0.748 (±0.050)	0.712 (±0.057)	0.641 (±0.056)	0.620 (±0.057)	0.743 (±0.052)	0.701 (±0.051)
Balanced accuracy	0.841 (±0.028)	0.807 (±0.031)	0.801 (±0.029)	0.781 (±0.028)	0.831 (±0.031)	0.776 (±0.032)

The scores are reported as mean with standard deviation. The reported F1 score, precision, and recall are for the expired group. AUC: Area Under Curve, EN: Logistic regression with Elastic net regularization, HAE: Hybrid Autoencoder, RF: Random Forest, VAE: Variational Autoencoder.

### HAE latent representations are stemmed from predictive and non-predictive features

**Figure 4:**
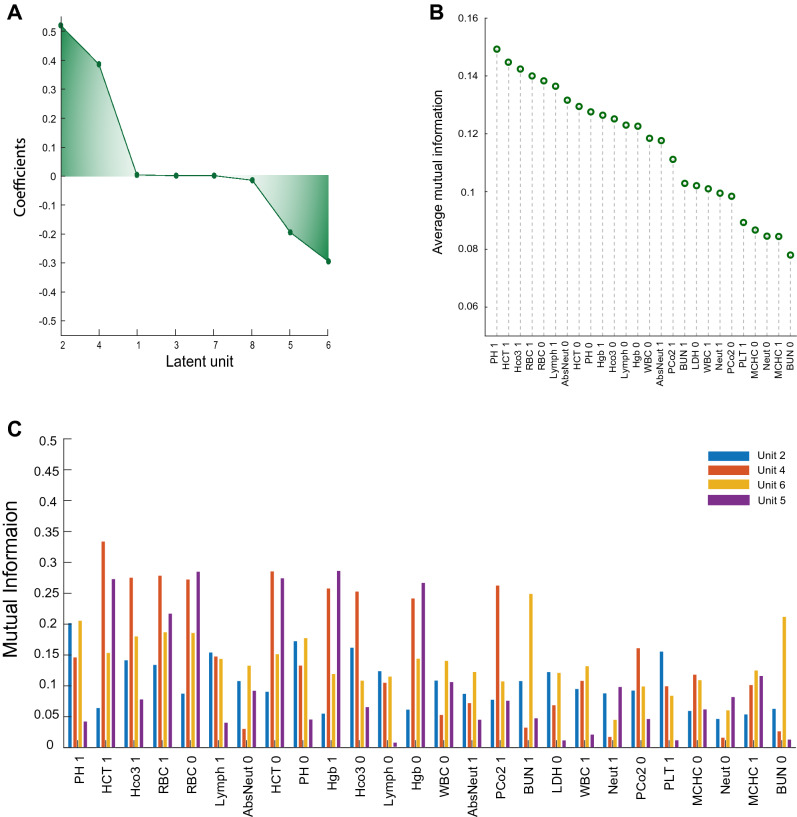
Mutual information of HAE latent representations**. (A)** The predictive weight of HAE latent units was obtained using logistic regression with L1 normalization. The green gradient visualizes the weight magnitudes. Units 2, 4, 5, and 6 had the highest weights. (**B)** Average MI between input features and latent representations. The values were calculated and averaged over all latent units. The plot displays all features whose average MI was equal to or greater than half of the maximum average MI. (**C)** MI from four units with the highest predictive power obtained from Panel A. Latent representations are dissimilar with respect to encoded input features. Besides features with medical evidence of being associated with COVID-19 mortality, such as the initial absolute neutrophil count, the predictive units also encode features that are not broadly known to be associated with disease prognosis, such as hemoglobin.

To investigate the characteristics of HAE latent representations, we compared the raw feature predictive weights obtained from the EN model with the importance of features that were encoded in HAE latent units with high predictive power. To do this, MI was utilized to find how much each latent representation was associated with an input. Logistic regression with L1 regularization was utilized to obtain four units with the highest absolute coefficient values (Fig. 4A). Average MI between latent units revealed the presence of 24 features whose MI was approximately equal to, or greater than, half of the average maximum MI. Compared with the EN feature weights, investigation of MI in HAE latent units with high classification power indicated that the units appeared to encode linear and nonlinear predictive power since the order of high MI features is dissimilar to EN, and several features, such as PH, have significant MI but low EN weights (Fig. 4B, Supplementary Figure 5). Furthermore, from a medical perspective, the HAE encodes both informative and non-informative features (Fig. 4B,C). For instance, the absolute neutrophil count (AbsNeut) is an important predictive feature in both the raw EN model (Supplementary Figure s5) as well as in predictive HAE representations (Fig. 4C). Previous studies have reported the association between COVID-19 prognosis and neutrophile count. In contrast, the WBC count had little weight in the EN model but was prominent in predictive HAE representations. Lastly, the RBC values had little predictive weight in the raw EN model but were prominently encoded in predictive HAE representations. This observation is most likely due to the regularization of MSE loss that forces units to also encode information for input reconstruction. Lastly, for many features, average MI across HAE latent representations revealed that the second instance of a lab result (e.g., PH 1) received more encoding than the first one. This observation is potentially related to the fact that maintaining homeostasis is a priority goal for the human body. Pathophysiological insults which cause an abnormal increase or decrease in biomarkers are often associated with elicited compensatory mechanisms, which attempt to resolve the insult and tune the biomarkers to the normal range^27^. Therefore, compensatory mechanisms limit the predictive information provided by biomarkers as time passes from a pathophysiological incident.

## Discussion

The current paper presents a hybrid framework for medical feature engineering to predict COVID-19 mortality prognosis from clinical, demographic, and laboratory features using variational autoencoders with triplet loss. The latent representations obtained from HAE provided reasonable classification performance with EN and RF models, and distinct patient clusters were observable in the PCs of HAE representations (Fig. 2C). Moreover, classifiers based on HAE latent representations demonstrated matched or better performance compared to those based on modified VAE and raw features (Fig. 3, Supplementary Figure s3, Supplementary Figure s4, Table 3, Supplementary Table s3). Also, Bayesian optimization was significantly faster in classifiers that utilized latent representations. Finally, MI between latent representations and features in the HAE model indicate that the representations with high predictive power encode both linear and nonlinear predictive features as well as both predictive and non-predictive features.

Autoencoders are advanced, unsupervised dimensionality reduction techniques that are widely utilized in various fields. Scalability, unsupervised nature, and ability to extract nonlinear relations make the use of these frameworks popular^28^. The accelerated and unstructured growth of recorded medical data from various sources, such as patient symptoms and laboratory data, results in large and complex datasets. The use of these data, therefore, becomes more complicated due to the presence of noisy and redundant features and inflated data volumes. For instance, as data dimensions increase, the "curse of dimensionality" becomes more significant, resulting in decreased efficacy and increased computational demands^29^. Increasing the input feature dimensions could initially improve classification performance. However, it has been observed that an extensive increase in data dimensionality beyond the "sweet spot" often causes a decline in model performance^30^. To address the aforementioned issues, dimensionality reduction techniques are used to uncover mappings that project data from high-dimensional raw space to a latent low-dimensional structure^31,32^. Decreasing data dimensionality is beneficial in many ways, such as reducing computation time, the removal of noisy and irrelevant data, and improving classification efficacy^33^.

A core issue in medical data dimensionality reduction approaches is the extraction of efficient, informative latent representations for subsequent classifications. Routine dimensionality reduction techniques used in classification tasks, such as PCA and t-distributed stochastic neighbor embedding (t-SNE), are rigid in the sense that they either focus on linear projections or cannot be directly tuned to extract informative representations depending on the question at hand^15^. The proposed HAE framework addresses this important issue by incorporating the Triplet loss function for unsupervised dimensionality reduction. Triplet loss leverages the assumption that members of the same class should be spatially close in the projected representation space. The algorithm adds a loss that aims to cluster data instances from the same class together in the representation space, enabling the approach to tune the latent representations based on the research question. Indeed, our results showed that models using VAE latent representations without Triplet loss had worse performance than the raw feature models in a COVID-19 prognosis prediction framework. In contrast, the HAE model had performance metrics that were on par or better than the raw feature models, showing that HAE was able to efficiently map raw input features to an informative lower-dimensional structure.

Previous studies have utilized AEs as parts of predictive model pipelines on various aspects of the COVID-19 pandemic, from epidemiology to hospitalization course and outcome prognosis. While most studies used imaging data, such as lung CT scans, several studies also utilized non-image tabular data for output prediction^34^. However, none of these studies implemented Triplet loss for autoencoder training and latent representation extraction using tabular data. A study by Leung et al. used a neural network model along with AEs for forecasting hospitalization outcomes, including ward admission, ICU admission, semi-ICU condition, and outpatient. The study reported decent performance of their model using non-image laboratory data from open-source data repositories^35^. Another study by Khozeimeh et al. combined Convolutional Neural Networks (CNNs) with AEs for predicting the mortality prognosis of COVID-19 patients. The authors reported a satisfactory predictive performance using tabular clinical data and binarized laboratory results (i.e., normal vs. abnormal)^36^. Jang et al. implemented a model using combined LSTM-autoencoder modules to forecast the number of daily admitted COVID-19 cases in Canada. The study was framed as a time-series prediction due to the nature of the task. The results showed that the proposed framework was superior to statistical time-series analysis and routine deep learning models^37^.

HAE latent representations were able to provide balanced predictions while reducing the optimization time of the implemented classifiers. In contrast to the common view that algorithms must replace human decision-making, a more robust approach is to augment human decisions with meticulously designed and tested algorithms^38^. Clinical implementation of ML models requires the establishment of trust between physicians and ML researchers to demonstrate the actual potential of the models. Moreover, ML models should be tuned and optimized based on their target implementation environment to avoid unintended consequences^39^. While many studies prefer to only report ROC curves, AUCs, and F1 scores, independently, these metrics are not enough to evaluate an ML framework. The precision-recall trade-off should be tuned based on the desired model implementation environment^40^. Accordingly, the current model was designed to be implemented in a hospital environment with limited computational resources to predict COVID-19 mortality prognosis using routine medical and laboratory features. However, the code can be tuned to predict any categorical outcome from the provided tabular medical data. This framework can be especially valuable for countries with lower socio-economic status, fewer resources, and limited healthcare personnel to augment routine clinical decision making towards a personalized medicine framework based on each patient's features.

A powerful aspect of the current HAE framework is its potential to be integrated into a hospital's database as a real-time framework to assimilate data from new patients and provide online predictions. AEs and other deep-learning frameworks are capable of online learning to avoid retraining the model when new data is added to the model^41^. Moreover, the potential of DL models for GPU implementation significantly increases their performance as the volume of training data increases^42^. With the increased computational power of modern computers and their integration in medical environments, the HAE model can be straightforwardly implemented on a hospital server to efficiently reduce the dimensionality of the incoming data for a final classifier algorithm. This approach significantly reduces the optimization time of the final classifier, as observed in our results, increasing the speed of training and prediction.

ML Algorithms are able to extract patterns from large volumes of provided data within the frameworks of a defined mathematical goal and metric^43^. Human decisions can be widely biased by physiological and psychological factors^43^. For instance, during the COVID-19 pandemic, fatigue from large patient loads, along with stress and fear from facing an unfamiliar pathogen, leads to many instances of erroneous decisions^44^. However, expert physicians are still more powerful than algorithms in making decisions based on a vast pool of experience, considering not only the clinical status of the patient but also drawing from the previous cases that the physician faced. To augment the decision-making of physicians, the employment of the HAE feature engineering framework in a hospital environment allows for efficient, rapid processing of large volumes of medical data in a reasonable time to provide predictions to help the final judgment of the physician.

Imaging modalities provide valuable diagnostic and prognostic information for various diseases. Chest CT scans can detect COVID-19 lung pathological alterations even in the early stages of the disease. Radiological markers indicating worsening and healing condition, such as presence of dense and diffuse consolidations and their resolution, have been highlighted by previous studies^45,46^. To harvest the information provided by imaging modalities, CNNs have been frequently utilized^47^. A prominent challenge is often to combine information from imaging modalities with laboratory and clinical data to maximize information utilization. Studies have used separate frameworks to first extract information from imaging data and then integrate them with clinical information^48^. Separate frameworks, however, increase the chance of coding bugs by making the pipeline more complex. A possible solution could be the usage of a framework similar to HAE to convert imaging data to latent representation with reduced dimensionality, and combine these with representation from clinical and laboratory data in a final classifier. Since the HAE framework can be used for feature engineering of both imaging and non-imaging data by changing the layer characteristics, the approach can simplify the merging of medical information for final insertion into a predictive model. Furthermore, new data points can be created from the HAE model since it is a generative model in nature. Compared with routine approaches like upsampling, the data points created using the HAE framework are potentially more useful for training purposes since they do not simply copy the training instances but use latent distributions that were extracted from underlying distributions.

Deep learning models are, by nature, considered to be interpretational black boxes. However, various techniques, such as the utilization of MI, can be helpful in making the framework more transparent. Due to the architecture of its loss function, the HAE encodes both predictive and non-predictive features. Therefore, relevant background knowledge is necessary for distinguishing the biologically plausible predictive features^49^. For instance, WBC, neutrophil count, LDH, and RBC levels were among the features with relatively high average MI. In contrast to limited evidence regarding the association between RBC levels and mortality prognosis^50^, many previous studies have demonstrated that a severe COVID-19 conditions presents with increased white blood cell (WBC) and neutrophil counts^51^. Neutrophils are the first responders to the invasion of pathogens and tissue damage mediating pathogen elimination using oxidative burst and phagocytosis^52^. In vulnerable individuals, neutrophilia could exacerbate the host immunopathological response, resulting in a more severe form of COVID-19^53^. The autopsy of expired patients' lungs has revealed neutrophil infiltration in pulmonary capillaries, extravasation into the alveolar spaces, and neutrophilic mucositis. Furthermore, increased levels of neutrophil extracellular traps (NET) in COVID-19 contribute to the occlusion of pulmonary micro-vessels, resulting in severe organ damage^52,54^. Lactate dehydrogenase (LDH), as an intracellular enzyme, plays a role in anaerobic glycolysis. Elevated serum LDH levels are associated with poor prognosis in various diseases, such as malignancies. In severe COVID-19 patients, elevated serum LDH level is an independent risk factor associated with severity and mortality^55^. Elevated LDH could reflect tissue injury caused by SARS-CoV-2, especially lung injury since it has been observed during both alveolitis and lung fibrosis^56^. Moreover, a strong immune response to a COVID-19 infection and following cytokine storm could also cause multi-organ damage, further elevating LDH levels^57^.

## Limitations and future works

The results of this study should be interpreted in light of several limitations. This study was carried out with retrospective data. Therefore, supervision during data collection was not possible to increase the quality of documentation during patient admission. Researchers were not blind to outcomes. The hospital had more severe and expired patient ratios than the overall Iranian society's ratio since it was a primary care center for COVID-19. Thus, the severity and mortality rates of this study do not reflect the population rates of these variables. As the patients were admitted during the first wave of the pandemic, the previous COVID-19 infection history was not considered a possibility and was not recorded by hospital physicians. It is likely that this factor had a limited effect since the first wave's patients were unlikely to have previously been infected by COVID-19. Imaging features were not utilized in the current study. Future studies could implement the HAE framework on combined image and non-image data to investigate possible performance improvements for prognosis prediction. Furthermore, larger, and more diverse study populations should be used for further evaluation of our findings. In this study, a binary outcome (i.e., discharged and expired) was used for prognosis. Prospective projects can focus on other outcomes, such as whether a patient was intubated or admitted to ICU. Finally, the framework of this study could be tested in other acute respiratory infectious diseases to investigate the efficiency of our model.

## Conclusion

The COVID-19 pandemic highlights the need for augmenting a human-centered healthcare system and decision making. Machine learning frameworks can enhance healthcare capabilities by finding patterns in large data volumes that usually escape the human eye. A major part of almost all machine learning frameworks is feature engineering. The current paper proposed a hybrid autoencoder (HAE) framework utilizing the features of variational autoencoders (VAE) along with MSE (Mean Squared Error) and Triplet loss to extract informative latent features for prognosis prediction using a main dataset and a public dataset for external validation. For performance evaluation, latent representations from the HAE and modified VAE frameworks and raw features were inserted into a final linear and nonlinear classifier, Logistic regression with Elastic Net regularization and Random Forest, respectively, to predict COVID-19 mortality prognosis from laboratory and clinical data. Classifiers using HAE latent representations displayed reasonable and balanced performance in comparison with the modified VAE representations and raw features. Furthermore, the Bayesian optimization time for the classifier utilizing the HAE latent representations was shorter than that of the raw features. Finally, MI between input features and HAE latent representations revealed that predictive representations coded both linear and nonlinear predictive features as well as, from a medical perspective, informative and non-informative ones. With the vast potentials of autoencoders, such as their ability for online learning and engineering of image and non-image data, the HAE framework of this study can be utilized in hospitals for online feature engineering and dimensionality reduction of the medical data with tuning based on the desired implementation scenario.

## Supplementary Information

Supplementary Information.

## Data Availability

The supporting data are available from Shohadaye Tajrish Hospital, Shahid Beheshti University of Medical Sciences, Tehran, Iran. However, restrictions apply to the availability of the data, which were used under permission for the current study, and so are not publicly available. Authors can provide the data upon reasonable request and with permission of the Ethics Committee and the information security office of the Shahid Beheshti University of Medical Sciences.
