# Supplementary material for: Hybrid feature engineering of medical data via variational autoencoders with triplet loss: a COVID-19 prognosis study
Source: Sci Rep. 2023 Feb 17;13:2827. doi: 10.1038/s41598-023-29334-0 (PMC9936112; doi:10.1038/s41598-023-29334-0)
Supplement: Supplementary file 1 — Supplementary Information. [file 41598_2023_29334_MOESM1_ESM.pdf]

Hybrid Feature Engineering of Medical Data via Variational Autoencoders with Triplet Loss: A COVID-19 prognosis study

Mahdi Mahdavi [1, 2 +], Hadi Choubdar [1, 2 +], Zahra Rostami [1], Behnaz Niroomand [1], Alexandra T. Levine [3], Alireza Fatemi[4], Ehsan Bolhasani [5], Abdol-Hossein Vahabie [6,7,8], Stephen G. Lomber [2] and Yaser Merrikhi [2]

[1]Department of Medicine, Shahid Beheshti University of Medical Sciences, Tehran, Iran

[2] Department of Physiology, McGill University, 3655 Promenade Sir William Osler, Montreal, QC, H3G1Y6, Canada.

[3]Department of Psychology, University of Western Ontario, London, Ontario, N6A 3K7, Canada.

[4]Department of Internal Medicine, Shohadaye Tajrish Hospital, Shahid Beheshti University of Medical Sciences, Tehran, Iran

[5]Department of Physics, University of Isfahan, 81746-73441, Isfahan, Iran.

[6]Cognitive Systems Laboratory, Control and Intelligent Processing Center of Excellence (CIPCE), School of Electrical and Computer Engineering, College of Engineering, University of Tehran, Tehran, Iran

[7]Department of Psychology, Faculty of Psychology and Education, University of Tehran, Tehran, Iran

[8]Pasargad Institute for Advanced Innovative Solutions (PIAIS), Tehran, Iran,

[+]These authors contributed equally to this work

Corresponding Authors:
Drs. Yaser Merrikhi (yaser.merrikhiahangarkolaee@mcgill.ca) and
Stephen G. Lomber (steve.lomber@mcgill.ca)

Department of Physiology
McGill University
McIntyre Medical Sciences Building
3655 Promenade Sir William Osler
Montreal, Quebec H3G 1Y6
Canada

Supplementary Table 1. List of input features and their descriptions

Category	Feature	Definition
Demographic	*Age*	Age of the patient; calculated from the patient's date of birth.
	Gender	Gender of the patient (male, female)
Clinical symptoms	*Shortness Of Breath*	Extracted from medical history upon admission
	Sputum	
	Hemoptysis	
	Chills	
	Cough	
	Fever	
	Headache	
	Sore Throat	
	Dizziness	
	Stomachache	
	Nausea	
	Vomit	
	Diarrhea	
	Body Pain	
Comorbidities	*Diabetes Mellitus*	This information was extracted from the past medical history that was recorded from the patient by a hospital physician.
	Hypertension	
	Cardiovascular Disease	
	Airway Disease	
	CVA	
	Cancer	
	Renal	
	Liver	
	Corticosteroid	
	Chemotherapy Drugs	
	Immunosuppressant Drugs	

Vital Signs	*Blood Pressure max*	The blood pressure of patients was assessed using the hospital's electronic BP monitors
	Blood Pressure min	The blood pressure of patients was assessed using the hospital's electronic BP monitors
	Pulse Rate	The pulse rate of the patient. Measured using an electronic monitoring device
	Respiratory Rate	Respiratory rate. Recorded by a physician as the count of patient respirations in one minute
	SpO_2	The blood oxygen saturation of patients was measured in the room without oxygen support using the hospital's pulse oximeters.
Habitual History	*Smoker*	Extracted from medical history upon admission
	Addiction	
	Alcohol	
Complete Blood Count	*WBC*	Obtained from venous blood samples analyzed in the central laboratory of the hospital
	Neutrophil	
	Lymphocyte	
	Mono	
	RBC	
	HGB	
	HCT	
	PLT	
	MCV	
	MCH	
	MCHC	
	ESR	
	CRP	
Coagulation	*PT*	Obtained from venous blood samples analyzed in the central laboratory of the hospital
	PTT	
	INR	
Biochemistry	*BUN*	Obtained from venous blood samples analyzed in the central laboratory of the hospital
	Cr	
	AST	

	ALT	
	ALkP	
	LDH	
	CPK	
	BS	
	CKMB	
Electrolytes	*Na*<br>*K*	Obtained from venous blood samples analyzed in the central laboratory of the hospital
Venous Blood Gas	*PH*<br>*PCO2*<br>*PO2*<br>*HCO3*<br>*BE*<br>*O2sat*	Obtained from venous blood samples analyzed in the central laboratory of the hospital

Supplementary Table 2. List of model input features

Category	Features	Category	Features	Category	Features
	Age		WBC_0		BUN_0
	Gender		WBC_1		BUN_1
**Demographic and Clinical Symptoms**	Cough		Lymph_0		CPK_0
	Fever		Lymph_1		ALT_0
	Dizziness		PLT_0		Creatinine_0
	Nausea		PLT_1		Creatinine_1
	Blood Pressure Max		ESR_0		ALkP_0
	Blood Pressure Min		HCT_0	**Biochemistry**	AST_0
**Vital Signs**	Pulse Rate		HCT_1		Troponin Quantitative_0
	Respiratory Rate		Mono_0		CKMB_0
	SpO2		RBC_0		LDHSerum_0
	Diabetes Mellitus		RBC_1		Qualitative Troponine
	Hypertension	**Complete Blood Count**	MCH_0		PH_0
	Cardiovascular Disease		MCH_1		PH_1
**Comorbidities**	Cerebrovascular Accident		MCHC_0		O2sat_0
	Cancer		MCHC_1		O2sat_1
	Renal Disease		HGB_0		BE_0
	Chemotherapy Drugs		HGB_1	**Venous Blood Gas**	BE_1
	Immunosuppressant Drugs		MCV_0		PCO2_0
**Habitual History**	Smoke		MCV_1		PCO2_1
	Alcohol		Neutrophil_0		PO2_0
	K_0		Neutrophil_1		PO2_1
**Electrolytes**	K_1		Abs_neut_0		HCO3_0
	Na_0		Abs_neut_0		HCO3_1
	Na_1		Abs_lymp_0	**Coagulation**	PT_0

		Abs_lymp_0	PTT_0
		CRP Quantitative_0	INR_0

Supplementary Table 3. External validation performance metrics of classifier models

Metrics	Logistic Regression (HAE)	Random Forest (HAE)	Logistic Regression (Raw Features)	Random Forest (Raw Features)	Logistic Regression (VAE)	Random Forest (VAE)
Time	12.11 (±2.26)	25.71 (±3.93)	13.87 (±1.70)	27.95 (±3.12)	12.49 (±2.31)	26.06 (±4.14)
F1-Score	0.774 (±0.050)	0.776 (±0.052)	0.712 (±0.051)	0.703 (±0.043)	0.633 (±0.061)	0.642 (±0.063)
AUC	0.891 (±0.032)	0.890 (±0.034)	0.854 (±0.034)	0.855 (±0.035)	0.771 (±0.043)	0.770 (±0.040)
Precision	0.747 (±0.060)	0.760 (±0.060)	0.697 (±0.062)	0.696 (±0.052)	0.620 (±0.059)	0.604 (±0.061)
Recall	0.805 (±0.069)	0.800 (±0.072)	0.731 (±0.061)	0.714 (±0.060)	0.675 (±0.084)	0.651 (±0.085)
Balanced accuracy	0.820 (±0.042)	0.822 (±0.040)	0.770 (±0.041)	0.772 (±0.036)	0.720 (±0.050)	0.700 (±0.048)
Averaged precision	0.832 (±0.052)	0.834 (±0.043)	0.794 (±0.048)	0.796 (±0.051)	0.680 (±0.061)	0.673 (±0.058)

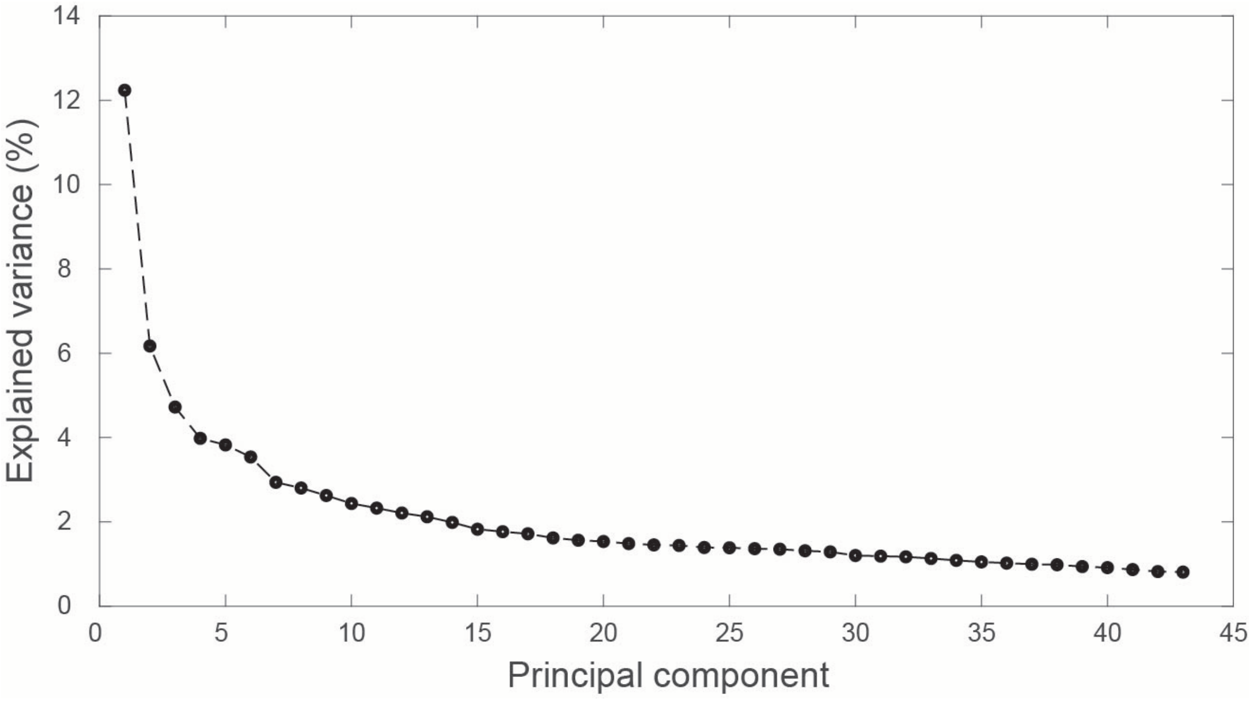

Supplementary Figure 1. Percent of variance explained by each principal component of raw features

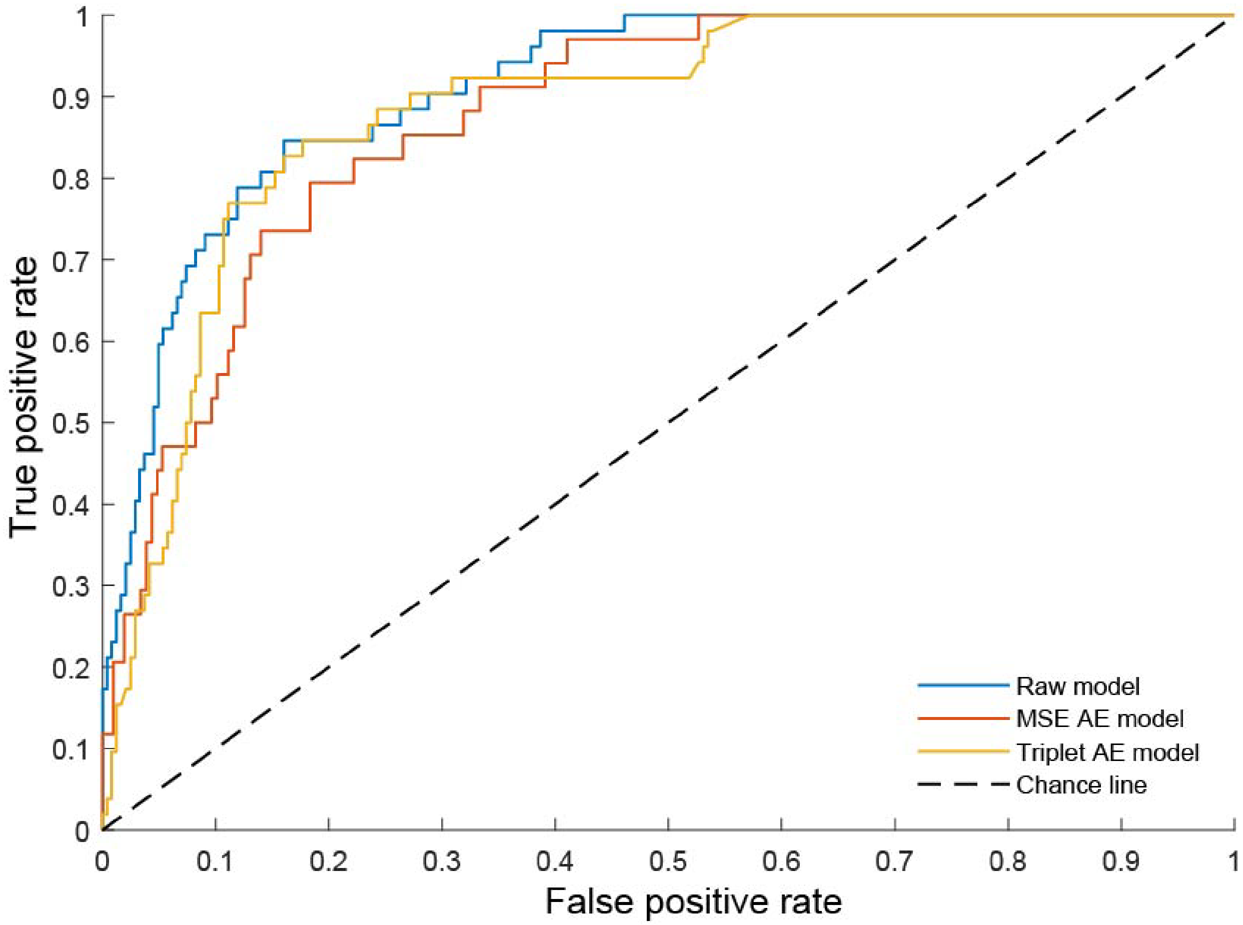

Supplementary Figure 2. ROC curve of the random forest model from raw features and representations from the modified VAE and hybrid AE.

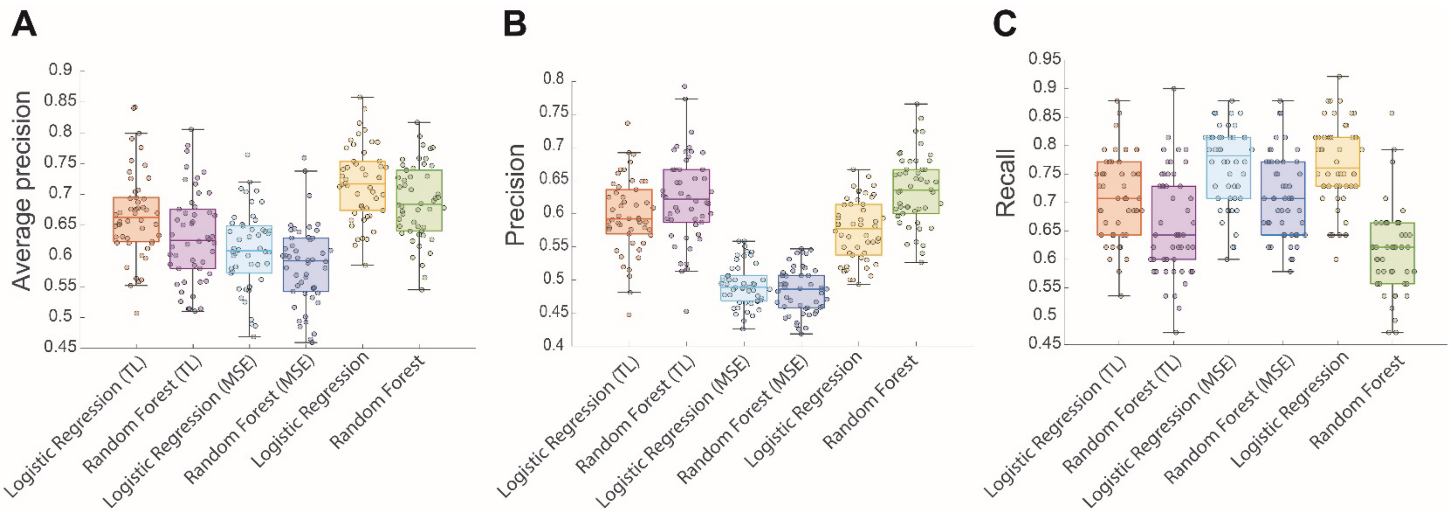

Supplementary Figure 3. Box plots of average precision (A), precision (B), and recall (C) of the classification models of raw features and representations from the modified VAE and hybrid AE. While the models using the representations from the modified VAE had high recall rates, their precision was markedly lower than those from the raw and HAE models.

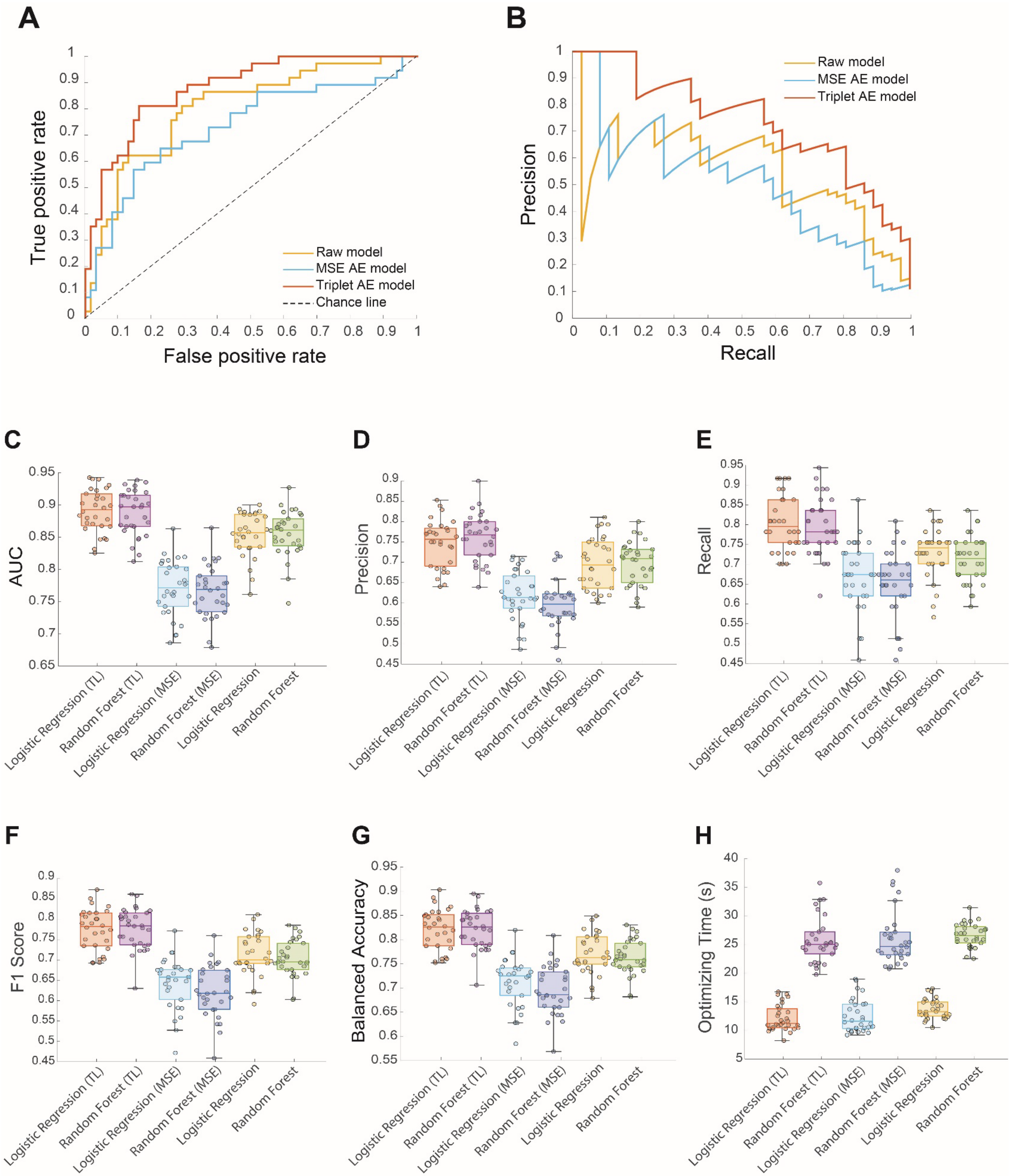

A
True positive rate
False positive rate
Raw model
MSE AE model
Triplet AE model
Chance line
B
Precision
Recall
Raw model
MSE AE model
Triplet AE model
C
AUC
D
Precision
E
Recall
F
F1 Score
G
Balanced Accuracy
H
Optimizing Time (s)
Logistic Regression (TL)
Random Forest (TL)
Logistic Regression (MSE)
Random Forest (MSE)
Logistic Regression
Random Forest

Supplementary Figure 4. Classification performance of raw features along with HAE and modified VAE representations. To enhance the generalizability of the study's pipeline, a public COVID-19 patient dataset from the Masih Daneshvari Hospital in Tehran was utilized for further validation. The input data consisted of 11 demographics, clinical, and comorbidity features, along with 26 laboratory features. The implemented pipeline was similar between the two utilized datasets and is extensively discussed in the methods section. **A)** ROC and **B)** precision-recall curves of the EN classifier. The HAE latent representations displayed better performance compared with latent representations from the modified VAE model and raw features. **C)** AUC box plots of the classification models. The AUC of the HAE model was higher than the raw and modified VAE models. **D)** Precision, **E)** Recall, and **F)** F1 score box plots of the classification models. The HAE latent representations were able to provide better and more balanced predictions with higher precision, recall, and F1 scores. **G)** Balanced accuracy box plots of the classification models. The balanced accuracy of the HAE model was higher than the raw and modified VAE models. **H)** Bayesian optimization time of the classification models. Compared with the full model, the HAE and modified VAE models required less time to be evaluated and optimized by the Bayesian optimizer. The optimization search spaces were similar for all three groups of input features. For panels C, D, E, and F, 30 iterations of training, optimization, and testing were utilized. TL: Models trained with representations from the HAE model; MSE: Models trained with representations from the modified VAE model.

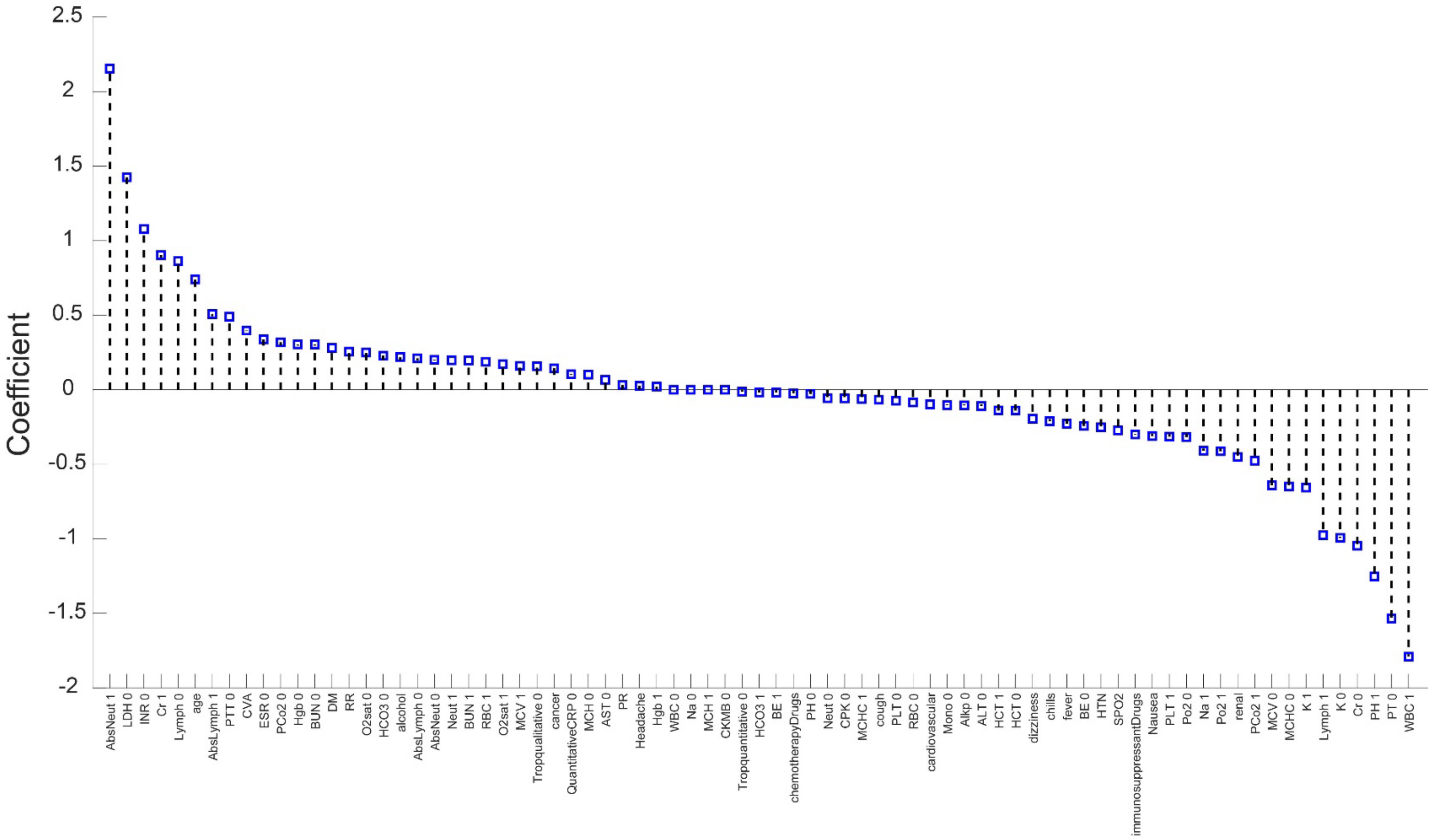

Supplementary Figure 5. Feature predictive weights from the logistic model with Elastic Net regularization.
